# Interdisciplinary human-centric architectural framework for One Health surveillance, early warning, and response: leveraging AI modelling and IoT environmental monitoring

**DOI:** 10.3389/fpubh.2026.1950115

**Published:** 2026-10-02

**Authors:** Abdulrahman Alzahrani, Hamoud Alshammari, Patty Kostkova

**Affiliations:** 1 Department of Information System and Technology, College of Computer Science and Engineering, University of Jeddah, Jeddah, Saudi Arabia; 2 Department of Information Systems, College of Computer and Information Sciences, Jouf University, Sakaka, Saudi Arabia; 3 UCL Centre for Digital Public Health in Emergencies (dPHE), WHO Collaborating Centre for Digital Public Health and Pandemic Preparedness, University College London, London, United Kingdom

**Keywords:** AI modelling, architecture framework, Internet of Things, One Health, vector-borne disease

## Abstract

Successful pandemic preparedness and sustainable healthcare security increasingly rely on One Health—linking humans, animals, and the environment. Novel real-time geolocated data sources from social media, the web, digital traces, and Internet of Things (IoT) sensors, combined with traditional surveillance data, are increasingly used to power AI models and enhance environmental sensing that is essential for preparedness and response in the One Health context. Despite the abundance of data streams and sensing data, the potential of AI and IoT environmental monitoring for One Health remains unfulfilled. The OHSEW (One Health Surveillance and Early Warning) framework architecture fills this gap by defining a human-centric architecture for One Health surveillance, early warning, and response, leveraging AI modelling and IoT environmental sensing, designed in particular for monitoring vector-borne diseases, zoonoses, and other environmental reservoirs of infectious diseases where the risk of spillover to human populations is high. In the OHSEW framework, we identify a rich spectrum of multiple heterogeneous data sources and a pipeline for improved surveillance, data visualisation, and training predictive AI models. These include open-source population and socioeconomic data, land use, satellite sensing, and weather forecasts, enriched by IoT environmental sensing (providing microclimate data streams) and traditional routine surveillance data reported by public health and environmental health experts. These models generate real-time data and actionable evidence for public health managers and policymakers, visualised through customisable dashboards, and provide early warning signals enabling rapid response and effective risk communication with the public. In this article, we outline the framework architecture, individual components, data pipeline, and configurable and localisable setup. To demonstrate the feasibility of the architecture, components of the OHSEW framework were piloted in real-world deployments of mosquito surveillance systems at two sites, Madeira, Portugal, and Cabedelo, Brazil. The architectures and results of these real-world deployments are presented to illustrate the feasibility of the OHSEW framework, with directions for further research outlined.

## 1 Background

In this section, we introduce the concept of One Health, disease surveillance, and early warning systems, as well as the related concept of antibiotic resistance (AMR), advances in AI modelling and Internet of Things (IoT) environmental sensing, and the unprecedented role of digital technology in addressing this global challenge (1).

### 1.1 One Health

The core of the One Health paradigm—linking humans, animals, and the environment—recognises the essential connections among animals, humans, plants, and ecosystems, as well as planetary health. One Health is the driving approach for detecting outbreaks of existing communicable diseases and potential spillovers (cross-species outbreaks) and for identifying settings of human-to-human transmission–the most likely onset of the next pandemic.

According to the World Health Organization (WHO), 60% of emerging infectious diseases reported globally come from animals, both wild and domestic. Furthermore, over 30 new human pathogens have been detected in the last three decades, 75% of which have originated in animals (2).

One of the highest threats is posed by vector-borne diseases (VBDs)—such as dengue, Zika, West Nile virus, and Lyme disease—as they account for more than 17% of all infectious diseases, causing more than 700,000 deaths annually, according to the WHO (3). They are further exacerbated by climate change, urbanisation, and global travel. VBDs transmitted specifically by mosquitoes in tropical parts of the world amount to an estimated 219 million cases globally and result in more than 400,000 deaths every year. From this perspective, mosquitoes are among the deadliest killers.

Health threats, vectors, and viruses do not respect borders. Only through intersectoral international collaboration, data and results sharing, and coordinated action can future outbreaks and epidemics be prevented and effectively controlled, early warnings of spillovers of new viruses from zoonotic diseases to humans expedited, and rapid response and effective management of diseases at local, national, and international levels enabled (4).

In order to achieve this ambitious goal, two major international activities have recently been established—the *Quadripartite* collaboration of UN agencies—Food and Agriculture Organization (FAO), United Nations Environment Programme (UNEP), WHO, and World Organisation for Animal Health (WOAH) (5). In Europe, the five European Union One Health agencies agreed on a similar strategic cooperation—including the European Centre for Disease Prevention and Control (ECDC), European Chemicals Agency, European Environment Agency, and European Medicines Agency (6).

In order to effectively advance the One Health agenda, monitoring of human and environmental systems needs to better analyse social interactions in society and interactions between humans and animals that might drive transmission of infectious agents from animals to humans and could result in a virus spillover. Therefore, a better understanding of societal dynamics and human–animal interactions across diverse settings and cultures worldwide is essential for strengthening preparedness and early warning systems. Furthermore, urban infrastructure, water management and sanitation systems, land use, and greenery also provide opportunities for vector breeding and enable interactions with humans. For example, a lack of potable water services and inadequate sanitation systems in low-resource settings in Brazil and other low- and middle-income countries (LMICs) create mosquito breeding hotspots (7).

Figure 1, illustrates the interconnections among human, animal, and environmental health.

**Figure 1:**
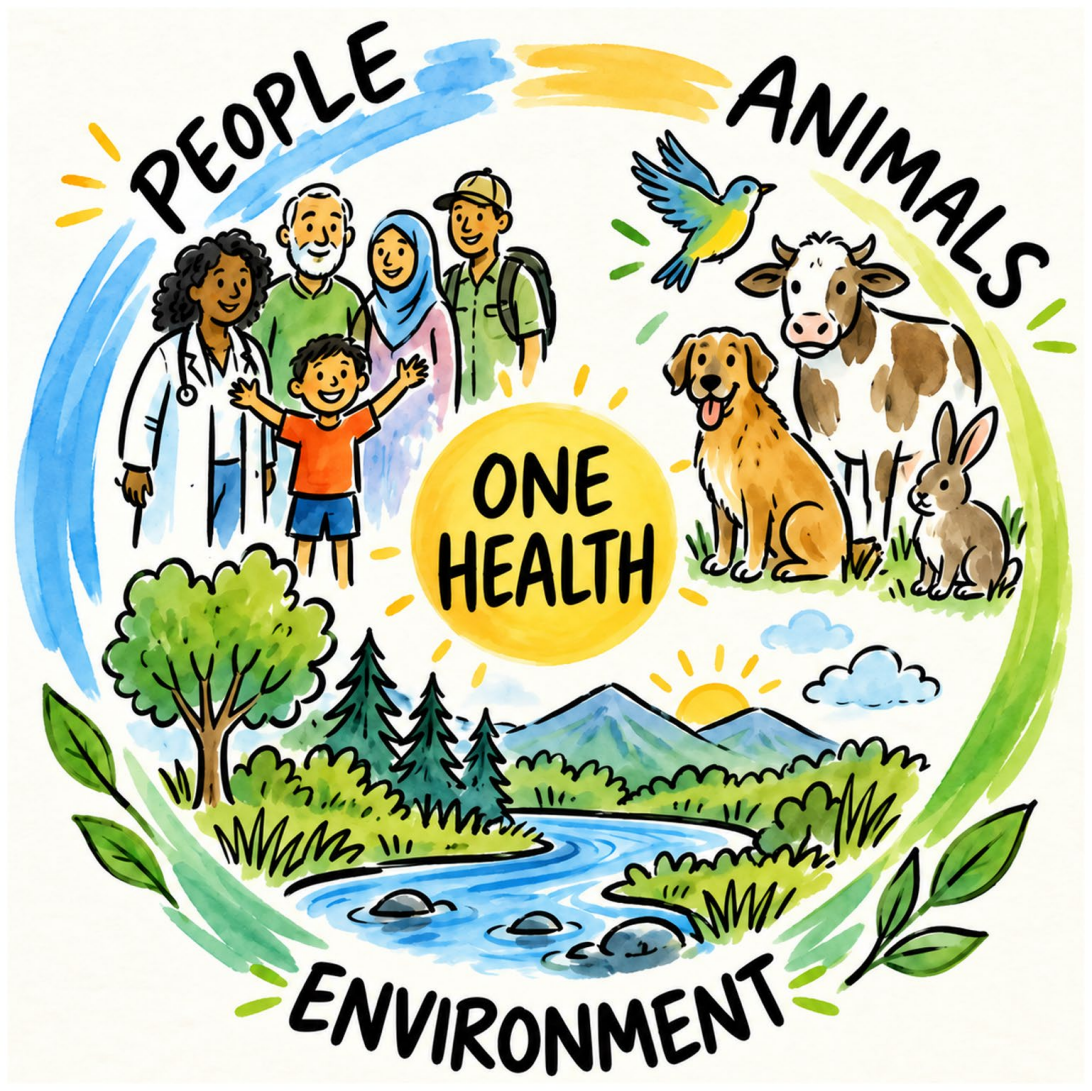
One Health paradigm (image: WHO).

### 1.2 One Health and Climate Change

Recently, One Health challenges have been dramatically exacerbated by climate change. Climate change has accelerated the emergence of pathogens and the resurgence of VBDs, in particular arboviruses, that are causing significant threats to both national and global health security (8). Changing rainfall patterns and increasing temperatures have recently contributed to public health outbreaks of dengue, Zika, Oropouche, and yellow fever in endemic areas and in new geographic territories that were previously not affected in Asia, Africa, and the Americas (9, 10), as well as increasing the risk of spillovers in a number of geographic regions (11, 12).

In the United Kingdom, the 2023 report on Health Effects of Climate Change (13) specifically addressed the impact of climate change on VBDs. In the United Kingdom, species capable of transmitting malaria and West Nile virus are already established. Imported dengue cases increased from 631 in 2023 to 904 in 2024, while chikungunya and malaria cases also increased (14). Owing to climate change, these vectors may soon become established in traditionally mild climates across Europe and Asia, leading to local transmission.

### 1.3 One Health and Antimicrobial Resistance

Antimicrobial resistance (AMR) is a major global challenge, often called a “slow pandemic,” that society faces because antibiotics are no longer effective against common infections. According to the 2019 study “Global burden of bacterial antimicrobial resistance in 2019: a systematic analysis” (15), it is estimated that drug-resistant infections contribute to nearly 5 million deaths every year, and AMR remains a major challenge in high-income countries such as Europe (16) as well as in LMIC countries (17).

AI models analysing genomic data from humans, animals, and environmental sources, such as wastewater, have demonstrated the potential for early warning and strengthening antimicrobial stewardship (AMS) (18). Furthermore, predicting resistance phenotypes from genomic data using supervised learning has also demonstrated potential to strengthen AMS (19).

### 1.4 Data in Disease Surveillance and Early Warning

Disease surveillance is a routine public health process that collects indicator-based data from clinical and primary care, microbiological laboratories, and the community to assess the burden of disease, support early warning and rapid response, and implement effective public health interventions. Public health “digital epidemiology” is a discipline that collects and analyses traditional indicator-based reported surveillance data from the healthcare system (GPs, clinicians, microbiological laboratories, etc.) and novel data streams and event-based surveillance information to expedite the routine process of data collection, inform public health authorities, and enable a rapid health response to the situation. The 2005 International Health Regulations (IHR) are designed to ensure that outbreaks of infectious diseases of international concern are reported to the WHO, enabling a timely response before they might spread internationally (20).

Early warning systems (EWS) provide alerts to public health experts when a signal is detected, and an event is verified as posing a risk of an infectious outbreak. Heterogeneous data sources in combination with indicator-based reported data are essential for driving an effective EWS (21). However, these come from a variety of sources—some are open-source data in the public domain, such as weather forecasting data (22), and some are from social media; others come from healthcare system surveillance and reporting, and some from local or country stakeholders—the data sources powering predictive models vary in quality, timeliness, and reliability throughout the epidemic progression of an outbreak (23). This is illustrated in Figure 2.

**Figure 2:**
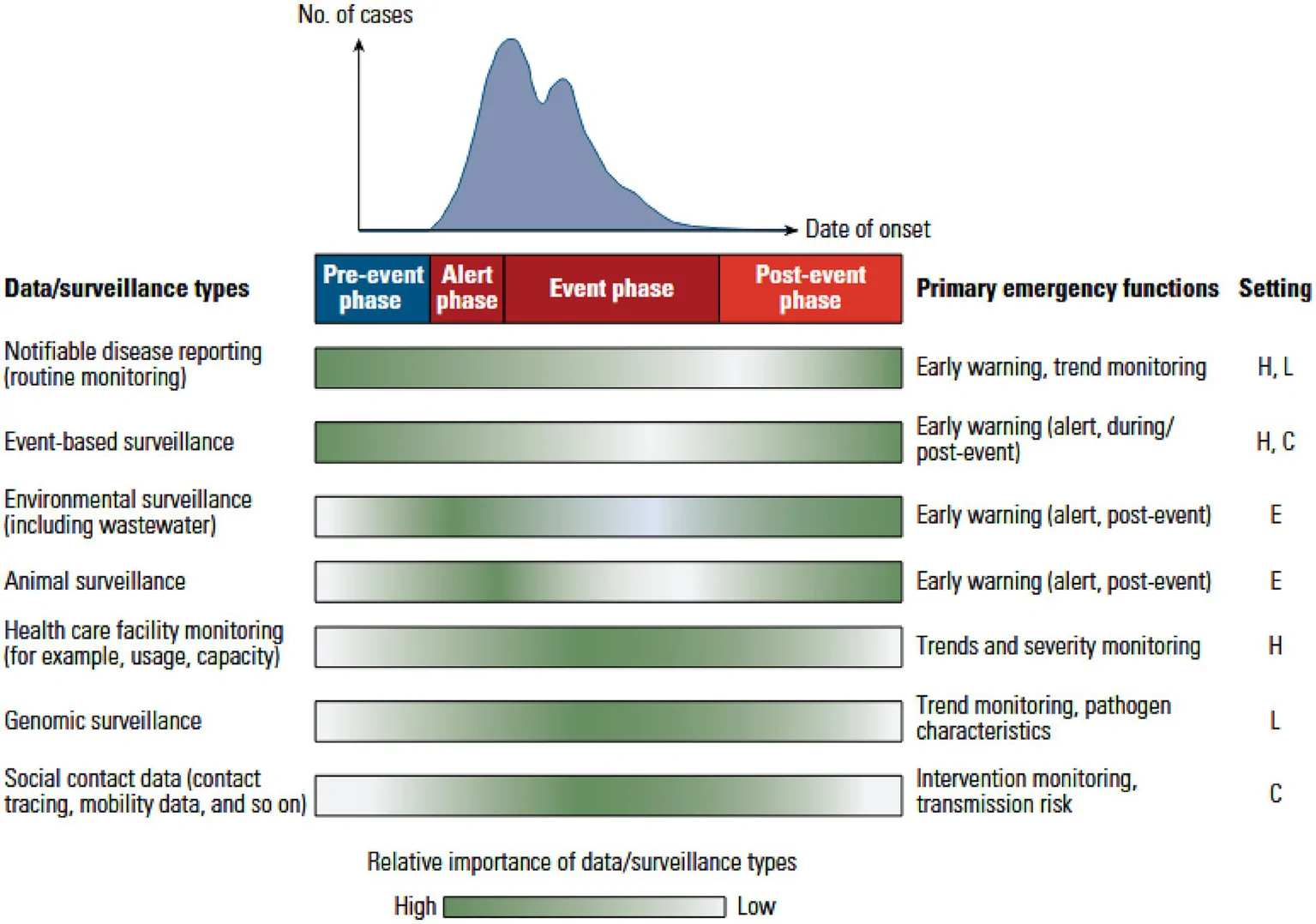
Variability and relative importance of different data and surveillance types for meeting emergency preparedness and response objectives over the course of an epidemic [fixture and caption from Archer et al. (23)]. The data and surveillance types listed are illustrative and non-exhaustive; many other approaches typically exist in countries. The assigned relative importance, primary functions, and settings relate to the outputs and decision themes that each surveillance type may be best placed to inform in or around an epidemic. Surveillance serves many other primary and complementary objectives for both routine monitoring and emergency response, and the relative importance of these objectives in any given phase varies widely across pathogens and contexts. C, community; E, environment; H, health facilities; L, laboratories.

EWSs fundamentally rely on the construction of predictive spatiotemporal models drawing primarily from indicator-based surveillance data sources and event-based surveillance information; however, they can also include data on weather, socioeconomic deprivation, historical climatic data, and local water infrastructure (24). Research into EWS has embraced AI but typically focuses on machine learning prediction performed “a posteriori” (25) to illustrate how the model would have performed if deployed before the outbreak. EWS based on datasets from official surveillance data or open data often lack the desirable granularity and timeliness needed to model the complex interactions between climatic and socioeconomic factors that are driving disease transmission (26). In order to improve the granularity and timelines, real-time reporting digital tools and apps for surveillance and reporting of human cases and vectors by public health and environmental experts, as well as by citizens through participatory reporting, are required. These bespoke real-time data streams developed for particular surveillance needs could then power decision dashboards and AI models for real-time reporting, model training, and model calibration.

*Epidemic intelligence* (EI) systems, such as Epidemic Intelligence from Open Sources (EIOS),^1^ have improved the speed of identifying new threats and events and enhanced risk assessment and rapid response through mining online news and social media for public health needs 24/7 in real time. For example, EIOS detected 80% of the public health events notified to WHO in 28 African countries, and 50% of events were detected earlier than officially reported to WHO (27). In addition to aggregated news platforms, Twitter emerged as an important social media platform during the swine flu pandemic in 2019 (28) and provided EI to numerous systems through its free search API for over a decade, including during the COVID-19 pandemic (29, 30). In 2023, free access to the Twitter API was discontinued as Twitter was bought by X (31).

However, EI and EWS need to be more widely implemented internationally in the One Health context to control zoonotic and VBD outbreaks, improve diagnosis, and strengthen risk communication with the public, farmers, veterinarians, and policymakers.

### 1.5 Internet of Things and AI Models for One Health

In addition to leveraging the indicator-based surveillance datasets and data streams discussed above, another opportunity to advance the One Health agenda is to deploy novel monitoring technologies such as the IoT (32). IoT sensors (optical, acoustic, odour-based, etc.) provide a promising opportunity for ecosystem monitoring to assess environmental conditions and disease or vector abundance in real time (33). IoT technology has been successfully implemented for air pollution monitoring to understand personalised exposure (34). However, the development and deployment of a low-cost IoT-based solution that could work remotely without a power supply, contain a reliable communication system (GMS, LoRaWAN, or Wi-Fi), robust microprocessors, and the appropriate combination of sensors for collecting data about humans, animals (through their sound or odour), and vectors (e.g., eggs laid) are still lacking (35).

While rapid AI advances are reshaping technological opportunities for automation, predictive modelling, surveillance, early warning, and rapid response (25, 36), tangible progress in public health and One Health has so far been limited (37–39). However, user-friendly AI tools have been shown to enhance environmental and disease modelling for EWSs, for example, for dengue (40) and malaria (41). It is important to keep human experts in control to ensure scientific robustness and equitable outcomes in the One Health context (42). Furthermore, opportunities and challenges of transfer learning (TL) and large language models (LLMs) deployed in healthcare systems have been summarised in a recent survey; however, this does not focus on One Health surveillance systems (43).

However, to further advance the One Health prediction of the entomological burden of diseases and vectors, AI-driven spatiotemporal models need to be trained on all the multiple data sources discussed above—human and animal surveillance data, laboratory data, social media and open data sources, climate and weather patterns, vector breeding locations, socioeconomic variables, landscape use, population behaviour patterns, and IoT environmental sensing data (39).

A number of studies have recently developed predictive AI models for EWS, as summarised in a systematic review (25). The projects focus on modelling individual diseases or vectors and do not provide a comprehensive unified framework. The review also highlighted that the models normally run on pre-collected datasets, are validated offline (“a posteriori”), and do not include support for routine data collection through dedicated digital apps and tools, which is championed by the OHSEW. Finally, an overview of AI models for climate-driven infectious disease outbreaks has been well summarised—highlighting the applications of AI models (such as support vector machines [SVMs] and random forests), deep learning methods (such as convolutional neural networks, long short-term memory networks, and transformers), and hybrid methods for capturing non-linear climate–disease interactions. However, the main challenges identified were data sparsity (in particular, limited data availability in low-resource regions), data quality, model explainability, and algorithmic bias (44). Furthermore, the review also does not include any study providing a comprehensive end-to-end architecture, nor does it propose overcoming the lack of data through the development of real-time, bespoke digital mobile surveillance tools and IoT sensors.

To conclude, a novel framework architecture is required to develop the entire data pipeline to collect, mine, and clean the data, train the predictive models, and disseminate results through user-friendly visualisation dashboards—providing real-time data to local governments and citizens, enabling evidence-based decision-making and rapid response. Such a One Health framework, leveraging routine surveillance-reported data, new data streams, and AI predictive models and incorporating real-time data from bespoke surveillance apps and IoT sensing technologies, is currently missing.

## 2 OHSEW Architectural Framework

The OHSEW architecture is an integrated human-centric (not human-in-the-loop) surveillance, AI modelling, and IoT sensing architectural framework for surveillance, early warning, and rapid response to One Health threats. The framework brings transformative tools to advance One Health through integrated surveillance, predictive analytics, and decision support, leveraging heterogeneous data in a unified manner and outlining interdisciplinary methodologies. As a one-size-fits-all system would not be able to serve all diseases and situations, the purpose of the OHSEW framework is to outline the architecture, essential data streams, and components for design, algorithm selection, and deployment challenges to consider when deploying the framework in specific situations. As the framework is modular, components could be selected and deployed as needed for each specific implementation of the architecture. This modularity is illustrated by the case study in Section 4.

Leveraging multiple heterogeneous data streams, such as mobile data collected by the routine surveillance and reporting by public health and environmental agents, IoT sensing streams, genomics, weather, climate, socioeconomic data, and animal and human surveillance cases, OHSEW recommends processing them through a standardised data pipeline and analytics algorithms to power visualisation dashboards for policymakers and spatiotemporal and AI models using data integration and pattern recognition to predict the burden of diseases and vector populations.

The key features of the OHSEW framework include the following:

1 The framework is generalisable and applicable to all diseases and vectors.
2 The framework has a modular architecture, with the following features:

- The six layers could be deployed as and when needed.
- Bespoke digital apps and tools support professional and public data reporting and have modular architectures for localisation.
- Reporting and predictive modelling tools are tailored to data sources and disease needs.
- Component-based dashboard modularity enables configurations, visualisation, and reporting for different levels of decision-making needs.

3 The framework incorporates real-time data from bespoke digital apps and tools for routine surveillance and reporting by professionals, as well as IoT environmental sensing data, to fuel dashboards, train predictive models, and calibrate their accuracy using ground-truth real-time data.

The core of the OHSEW framework consists of a versatile architecture for One Health surveillance, early warning, and response systems integrating the following six layers:

1. One Health Data Sources: This layer mines multiple datasets from syndromic indicator-based surveillance and real-time event-based sources from humans, animals, and the environment.
2. Data Integration through a Federated Architecture: This layer allows data to be kept at data providers and accessed via APIs as and when needed to preserve data privacy.
3. Spatiotemporal and AI Modelling for Early Warning: This layer includes a variety of statistical and AI methods for predictive modelling.
4. Component-based Configurable Dashboard: This layer provides visualisation and reporting of surveillance data, GIS mapping, and the results of AI models for public health experts and policymakers at all levels.
5. Interventions and Control Measures: This layer enables users to respond to public health threats at all levels.
6. Public Risk Communication and Community Engagement: This layer supports citizen awareness and local engagement.

Figure 3 illustrates the architecture and data-flow pipeline. First, data are collected from multiple sources, including human, animal, and environmental surveillance, including routine surveillance data from public health reporting, veterinary clinics, and farms, as well as data collected by bespoke digital apps and tools and IoT sensors from professionals and the public. In particular, bespoke digital apps and tools for professional reporting and IoT sensing are core components enhancing Layers 3 and 4 by replacing paper-based systems or manually reported data. IoT sensing data and open data complement the streams.

**Figure 3:**
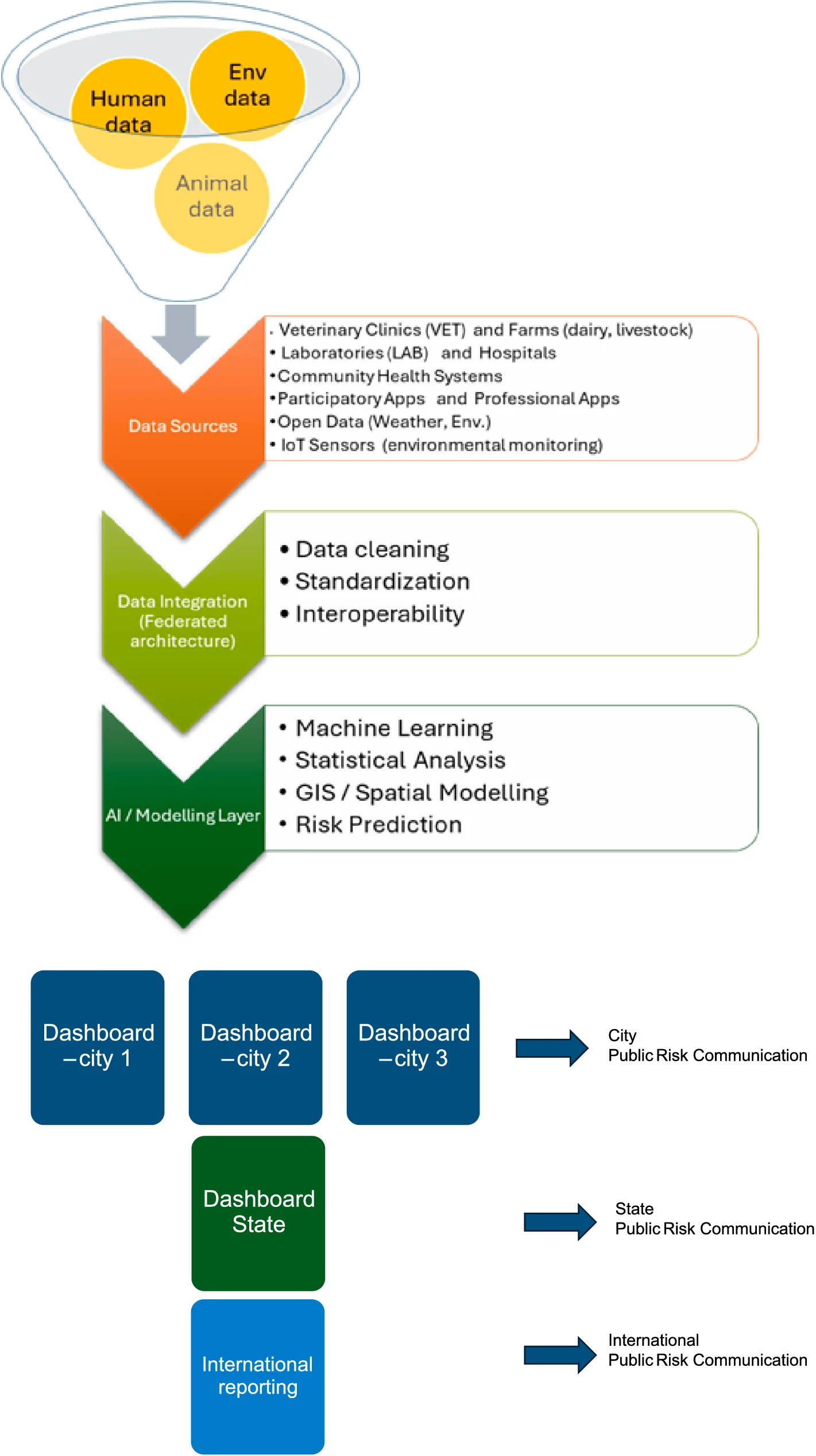
OHSEW architecture framework.

Second, data undergo cleaning and calibration, missing data are imputed, and middleware tools ensure interoperability through compliance with standards, while the federated architecture protects data privacy. Third, the modelling layer deploys statistical spatiotemporal and AI models to power customisable dashboards for reporting and early warning, configurable for appropriate levels of decision-making. The next sessions provide comprehensive descriptions of the layers.

The purpose of the architecture is to outline the high-level components, their features, principles, and data flows. As such, OHSEW could be customised and adopted for monitoring different vectors, utilising a variety of data sources at local, municipal, national, and international levels, and deploying only the components and layers required. This framework architecture is not aimed at presenting a fully specified platform but rather at outlining the customizable components and data pipelines essential for building a bespoke system, as a one-size-fits-all architecture would not capture the variety of diseases, local needs, and configurations. A customised OHSEW for VBD surveillance, modelling, and response, implemented using Layers 1–4, is presented in the next section to illustrate the real-world applicability of the framework.

### 2.1 One Health Data Sources

OHSEW is built on the integration of heterogeneous data sources. For each deployment, it is essential to start by considering what data streams are available for a given country and disease. It is recommended to consider developing and deploying bespoke digital data collection apps and tools, including IoT environmental monitoring systems, to improve the quality, granularity, and timeliness of data sources. The data streams might include the following:

(a) Integrating syndromic surveillance data from human and animal diseases, clinical records, laboratory diagnostics, genomic sequences, and data from monitoring the ecosystem and environment.
(b) Static, publicly available datasets, such as population density, socioeconomic index, land use, vegetation data, and water and sanitation infrastructure.
(c) A heterogeneous spectrum of real-time geolocated data streams, including event-based news, social media content, EI sources, digital traces, and weather forecasts.
(d) IoT-based environmental, animal, and wildlife sensing (acoustic, optical, and odour-based), satellite data, and climate variables, if available or developed for bespoke needs of the system.
(e) Bespoke apps and digital tools for professionals for surveillance and real-time reporting.
(f) Citizen and participatory surveillance through proactive, bottom-up reporting.

The data governance specifications and data privacy considerations are included in Layer 2. These should include data resolution and details on update frequency, as defined in Table 1. Case definitions and quality control rely on local staff training, calibration of public data, and imputation of missing data as required. Reliability is also defined for all variables, as outlined in Table 1.

**Table 1:** Classification of data streams for epidemic intelligence [expanded from Kostkova (46)].

Data Streams	T	R	I	A	G	S	ST
News Online	Days	Med	News	A	Y	N	N
Digital Traces	RT	High	Person	A	Y	Y	Y
Social Media	RT	Low	Low	A	Varied	Y	N
Routine Surveillance Data	Days/Weeks	High	Prof	Varied	Y	Y	Y
Pro-Med	Varied	High	Prof	H	Y	Y	N
Lab/Clinical Rep	Weeks	High	Prof	H	Y	Y	Y
Participatory Systems	RT/Says	High	Person	A	Varied	Y	Y
IoT Sensors	RT	High	Seamless	A	y	Y	Y
Weather Forecasts	RT	High	Prof	A	y	Y	Y

In the One Health context, as with human surveillance data illustrated in Section 2.4, these data streams also exhibit varying levels of quality, reliability, and timeliness (23). They have been classified according to these characteristics (46):

- T = timeliness (real-time, “days” after event, “weeks” after event).
- R = reliability (high reliability, medium reliability, low reliability).
- I = intention to share (personal, news, professional, or seamlessly collected).
- A = collection and mining by EI systems (automated, human-moderated).
- G = geolocation (yes, no, some).
- S = situation-aware (user or case context known, not known).
- ST = structured (yes, no).

### 2.2 Data Integration through Federated Architecture

Data coming from heterogeneous streams into the data pipeline need to undergo fusion and cleansing based on validation and verification results obtained when setting up the architecture for a specific OHSEW system, depending on the data types available.

Each data stream requires an independent bespoke cleaning and validation process based on the type of stream and frequency of changes, as illustrated in Figure 2 and Table 1.

For data fusion, data harmonization and standardisation using middleware software components should follow information coding and protocol standards (such as MESH/SNOMED CT, ICD, HL7/FHIR, and OIE-WAHIS) to allow further data reuse and interoperability with other systems.

Finally, for data storage and location, OHSEW recommends not storing all surveillance data in internal databases, which could be prohibited by national law, but rather accessing them via a federated architecture, preserving data privacy of the integrated data streams. These measures could be achieved through a well-defined threat model relying on a number of techniques: (1) agreement on transparent data formats and standards (such as those listed above) enabling interoperability, (2) data access rights through role and identity management, and (3) enforceable organisational agreements among stakeholders. Following these principles would contribute to making cross-site, privacy-preserving data access, model training, and validation safe and auditable. This could be achieved by accessing data through open API function calls exposing search results and data in cumulative anonymised formats. For example, while the framework operates on various levels, sensitive information, such as users’ symptoms and public health workers’ performance, important for decision-making and personalised response at community level, will only be shared with the state or national governments in cumulative formats as indicators of prevalence of cases and progress of surveillance efforts. Providing full transparency and data-sharing agreements is essential for gaining access to data and stakeholder trust.

### 2.3 Spatiotemporal and AI Modelling for Early Warning

The next step in the OHSEW data pipeline is the modelling component, which will be developed methodologically using a variety of techniques and their combinations—statistical and AI—depending on the disease or vector under study, data availability, opportunities to develop bespoke apps, digital tools, and IoT sensors, and, most importantly, the EWS needs defined by end users and key stakeholders. The methods might include the following:

- Spatiotemporal modelling: Time-series models incorporating climate variables (temperature, rainfall, and humidity) for predicting vector breeding and human cases. Bayesian spatiotemporal hierarchical regression using integrated nested Laplace approximation (INLA) can be used for predicting raster-based hotspots.
- Machine learning (ML): Supervised and unsupervised learning for classification, regression, clustering, and anomaly and outbreak detection.
- Deep learning (DL): Neural networks for image recognition (e.g., radiology, pathology, and wildlife monitoring), including deep neural network (DNN) methods and long short-term memory (LSTM) methods (47).
- Natural language processing (NLP): Mining textual data from surveillance reports, social media, news data streams, and citizen participatory apps.
- Geospatial AI: Integration of satellite imagery, GIS data, and climate modelling for hotspot prediction. The integration of remote sensing data with epidemiological records can enable spatial risk mapping.
- Network analysis: Modelling transmission pathways among humans, animals, and environmental reservoirs and predicting spillovers.

AI advances allow public health and One Health experts to build AI models without requiring programming expertise (48). As OHSEW is a generic, modular, end-to-end architecture, the appropriateness of model selection and modelling approaches for each particular OHSEW implementation will depend on the needs of the implementation, including the available datasets, country or setting, opportunities to develop bespoke apps and IoT systems for real-time data collection, and the diseases being surveyed. As illustrated in our case study in the next section, even with similar datasets from the same site, different methods might be appropriate (Section 4.3). This section outlines the opportunities, guidance, and key points to consider when implementing the OHSEW framework.

Model selection primarily depends on the target product profile (TPP), which determines the required resolution and how far ahead the EWS should provide alerts—both in terms of the spectrum between the minimal risk (acceptable floor) and optimal (aspirational target) criteria. For example, the prediction target could be defined as an excessive level of cases or risk (0 or 1) or as a fine-grained probability (0–100%), depending on the implantation of the OHSEW framework. Spatial units would be determined by the level of data collection, availability, and reporting opportunities (real-time, case or household level, neighbourhood level, district level, etc.). Furthermore, the frequency of data collection or aggregation (such as daily, weekly, or monthly) would be determined by local resources, staffing availability, reporting capacity, and response needs. Forecast horizons will be decided by the stakeholders and depend on the response actions required, ranging from seasonal vaccinations to real-time staff resource reallocation, depending on the disease, data available, and response feasibility.

The evaluation of the models again depends on the actual implementation system and needs; however, in general, model accuracy should be assessed using standard metrics, such as the root mean squared error (RMSE) to evaluate prediction error, precision and recall, F1 score (the harmonic mean of precision and recall), the confusion matrix, specificity and sensitivity, the receiver operating characteristic (ROC) curve, and area under the curve (AUC). However, these metrics could be misleading in spatiotemporal models.

Thus, probabilistic performance measures, such as the Brier score, should also be considered, including Murphy decomposition (reliability, resolution, and uncertainty) and calibrated intercept and slope via logistic recalibration *per block*, as models could be overconfident in some regions and underconfident in others. For prediction intervals, it is important to consider trade-offs between internal coverage and sharpness.

Furthermore, for external validation of models, the following should be considered: (1) *temporal ordering* (with purge or embargo windows (the longest lag or rolling window, as in our case study below)) and (2) *spatially continuous blocks* (given by the empirical spatial data range, with buffer strips excluded from training to avoid leakage). Thus, a combined design would ensure that each validation fold is separated from the training data by both a time embargo and a spatial buffer.

Ensemble methods (aggregating predictions from multiple prediction models) and continuous assessment using ground truth data from medical reporting or environmental sensing are useful for calibration to avoid model decay (49).

The spatiotemporal alignment strategy for fusing multisource spatiotemporal data (as defined in Section 3.1) will again be determined by the constraints and data availability of the actual implementation of OHSEW. For example, aggregation windows should be determined by the frequency of the data streams (typically, using sliding windows or anchored windows, as illustrated in the case studies in the next section). Depending on the data pipeline and fusion, future data leakage should be prevented by ensuring that source data have timestamps strictly earlier than the timestamp of the fused dataset. However, within the real-time recalibration framework proposed by OHSEW, the new data would be used to calibrate the results and added to the training dataset for iterative model runs. Lag selection in the context of One Health is predominantly determined by the professional expertise of the stakeholders.

AI provides opportunities to manage public health emergencies, offering not only significant advantages such as speed and personalisation but also risks due to accuracy and bias that could exacerbate health inequalities. Addressing these challenges requires regulation and societal collaboration (50) and should be considered when deploying OHSEW in particular situations.

It is also crucial to communicate AI model uncertainty and confidence measures (such as RMSE, F1 score, precision, and recall) to stakeholders and the public (depending on the end users) when developing the systems and through the dashboard, as the actions taken and risk communication methods will differ according to the audience needs. Risk communication techniques might include heatmaps for GIS visualisation, percentage-based reliability indicators using green, yellow, and red confidence levels inspired by weather forecasting, risk matrices, and other textual and visual methods (51).

Finally, the actual benefit of the EWS and its alerts needs to be determined for each implementation of OHSEW with key stakeholders using standard methods such as decision curve analysis (DCA) and utility-based evaluation (cost–benefit analysis) applied to local conditions, staff resources, disease transmissibility, and other local parameters.

### 2.4 Component-Based Configurable Dashboard

EWS-powered dashboards integrate multisectoral data streams into actionable insights for managers and policymakers. Typical dashboard components might include tables and graphs with cumulative numbers of cases, time series, GIS maps and visualisations, etc., depending on the needs of the actual system and the diseases or vectors under surveillance. Most importantly, dashboards should address the needs of managers and policymakers at appropriate levels—local, municipal, state/regional, national, and international—enabling truly evidence-based decision-making and rapid, data-driven responses.

Levels of decision-making and corresponding dashboard components might include the following:

- Local/community level: local case data, staff monitoring and workforce allocation, local hotspots, community engagement and local response, and local incentives and predictions.
- Municipality/city level: aggregated case data, multiple diseases, major hotspots, trends.
- Region/state level: statistical summaries, trends, GIS visualisation, government-level reporting, and predictions.
- International level: country-level reporting to organisations such as WHO, FAO, and WOAH.

In addition to visualising actionable information, dashboards also foster structured collaboration among key stakeholders—ranging from local community health and environmental agents to federal/national and international policymakers. In addition to the outputs of AI models and visualised data, human expertise is essential to calibrate the models, align surveillance protocols, streamline data collection to improve reporting and data harmonization, and establish cohesive national policies for evidence-based disease control. The dashboards serve all levels vertically while also interlinking experts and communities horizontally; therefore, OHSEW creates an integrated approach that ensures that surveillance data are systematically used to inform health interventions at all levels.

### 2.5 Interventions and Control Measures

The early warning results need to be followed by effective interventions and the implementation of policies and measures to control the outbreak. These interventions will be configured for each implementation of the OHSEW system depending on local needs and response policies and will be presented and recommended by the OHSEW EWS based in their suitability for the local situation, as defined by the public health experts at the deployment stage.

First, community interventions need to leverage behavioural patterns to enhance community actions and improve adherence to protective measures. These interventions could be digital, personal, or a combination of both and should primarily focus on high-risk communities with high disease or vector abundance identified by the OHSEW EWS.

Successful community interventions ought to be evaluated to assess their effectiveness and inform further action. Their outputs can then be integrated into the data streams collected and visualised in the dashboards for a seamless experience from the perspective of decision-makers.

Next, at the local public health level, OHSEW empowers local managers to implement more efficient staff allocation and planning strategies based on modelling of risks, disease spread patterns, and vector breeding hotspots.

Interventions could also be disease- or vector-specific. For example, Wolbachia mosquito population-control technology run by the World Mosquito Programme could deliver a targeted intervention in high-abundance areas identified by the OHSEW EWS to suppress breeding (52).

Finally, to best support decision-makers, intervention recommendations could include AI agent-based model simulations of species interactions, disease spread, and the expected impacts of protective policies and measures. Although this may be one of the most difficult components to implement realistically, these simulations could be of particular importance in time-sensitive decision-making processes, such as emergencies. The results could also be visualised in the dashboards for decision-making at all levels. An example of a prototype pandemic simulator leveraging the EIOS system is being developed by the WHO Pandemics Hub (53).

### 2.6 Public Risk Communication and Community Engagement

The next step is public risk communication, which informs citizens about risk levels, upcoming threats, and recommended public health policies and behaviours. This could be delivered through television, mass media, online and participatory apps, automated SMS or WhatsApp alerts, early warning notifications, and public-facing dashboards.

Tailored risk communication messages for emergencies need to reflect the level of risk to citizens at a given time and location, as well as the relevant cultural context, and be delivered in an appropriate language to improve understanding of and compliance with public health measures and extend their reach (54).

## 3 Pilot implementation—VBD mobile and IoT surveillance in Brazil and Madeira

With OHSEW framework providing the overarching One Health framework, this section demonstrates the system feasibility and implementation of the framework on VBD surveillance. In this section, we briefly describe the pilot as a case study to demonstrate the potential of the unified OHSEW system.

We implemented several OHSEW components that were deployed in real-word settings for mosquito-borne disease surveillance using mobile technology, IoT sensing, and managerial dashboards in two settings, Cabedelo, Brazil, and Madeira, Portugal, over a period of 2 years (55, 56). To preserve clarity and focus on the architectural framework, the details of the implementations and full results are beyond the scope of this study and can be found in the referenced publications. As the OHSEW architecture is a modular framework, it allows the selection and deployment of the layers and components needed for the actual implementation in real-world settings. In our case study, Layers 1–4 were deployed, while Layers 5 and 6 were not required and are subject to future work.

### 3.1 Design and Implementation

As we outlined above, VBDs account for more than 17% of all infectious diseases, causing over 700,000 deaths annually and making mosquitoes among the deadliest disease vectors. Despite the high disease burden, mosquito control remains largely manual, paper-based, fragmented, and poorly resourced, severely limiting evidence-based decision-making and rapid response by decentralised health governance systems in countries like Brazil and Portugal.

The One Health OHSEW system for VBD surveillance, developed through the MEWAR, MOISS, and MMSA projects, implemented four OHSEW layers, 1–4. Layers 5 (Interventions and Control Measures) and 6 (Public Risk Communication and Community Engagement) were not required at the time of deployment.

Also, following OHSEW feature 2b (bespoke digital apps and tools for professional and public data reporting with modular architectures for localisation), we developed two client applications: a bespoke digital app with a modular architecture for mosquito surveillance and a web-based manager module (dashboard), corresponding to OHSEW Layer 3.

To reflect the surveillance processes and user profiles (roles) of environmental health workers in Brazil and Madeira, the mobile app includes four modules: Ovitrap, Property, Laboratory, and Gamification. The system serves multiple user profiles: environmental agent, laboratory user (technician), operational manager, and strategic oversight (secretary of health and policymakers). As recommended by the OHSEW farmwork, the architecture enables site-specific deployment and localisation. In Madeira, Ovitrap and Laboratory modules were deployed, whereas in Cabedelo, Brazil, the Property module and Manager Dashboard were deployed. The architecture is illustrated in Figure 4 and is described in greater detail in (56).

**Figure 4:**
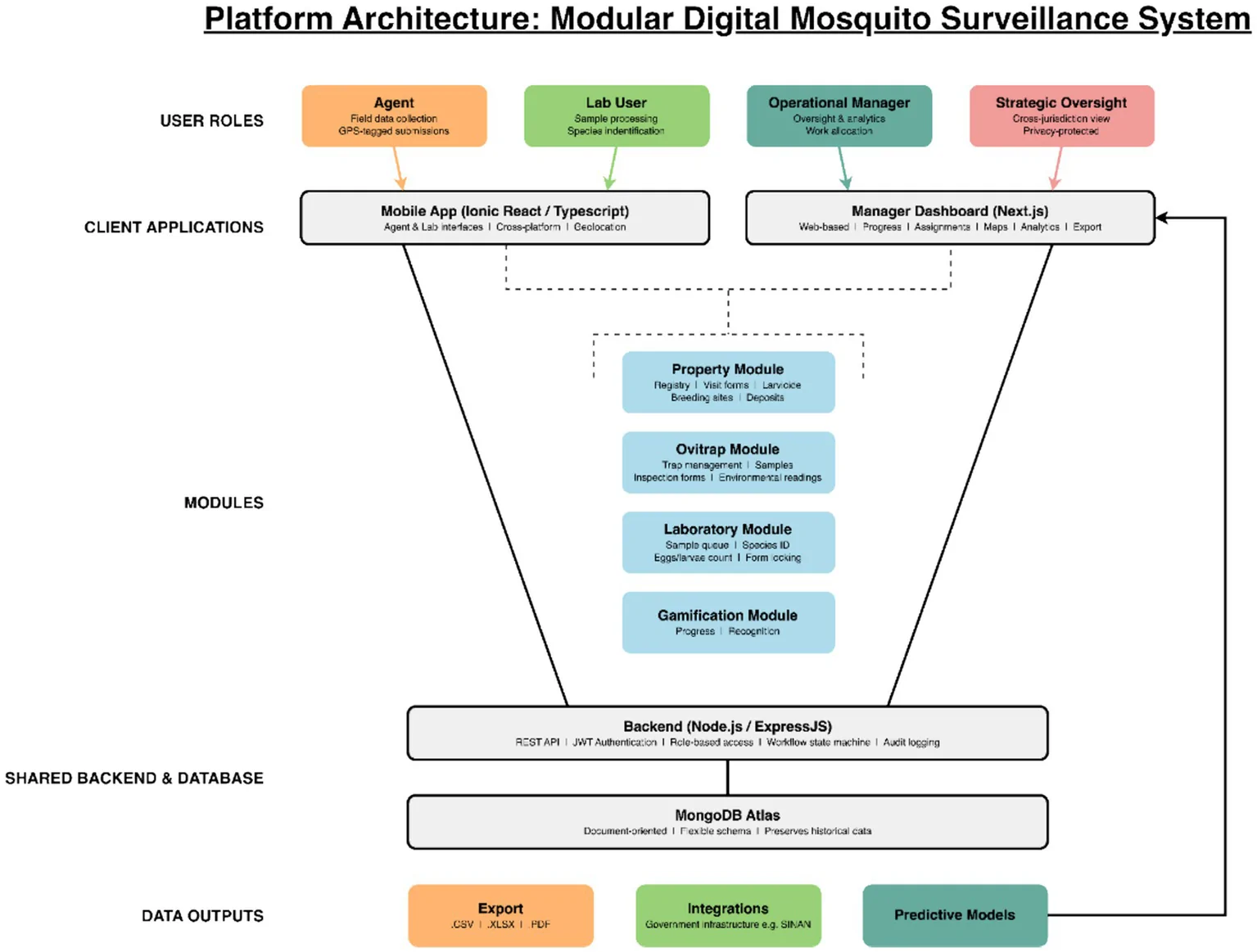
Platform architecture—modular digital mosquito surveillance system [from Raynolds et al. (56)].

Following OHSEW Layer 1, the system implemented real-time IoT water and weather sensor sets to monitor mosquito-breeding microclimate conditions in mosquito ovitraps. The system used an Arduino processor and GSM SIM card connectivity to transmit data in real time for AI-based predictive modelling of mosquito breeding, with the data stored on a Heroku server running a MongoDB database. This architecture is illustrated in Figure 5 and is described in detail in (56).

**Figure 5:**
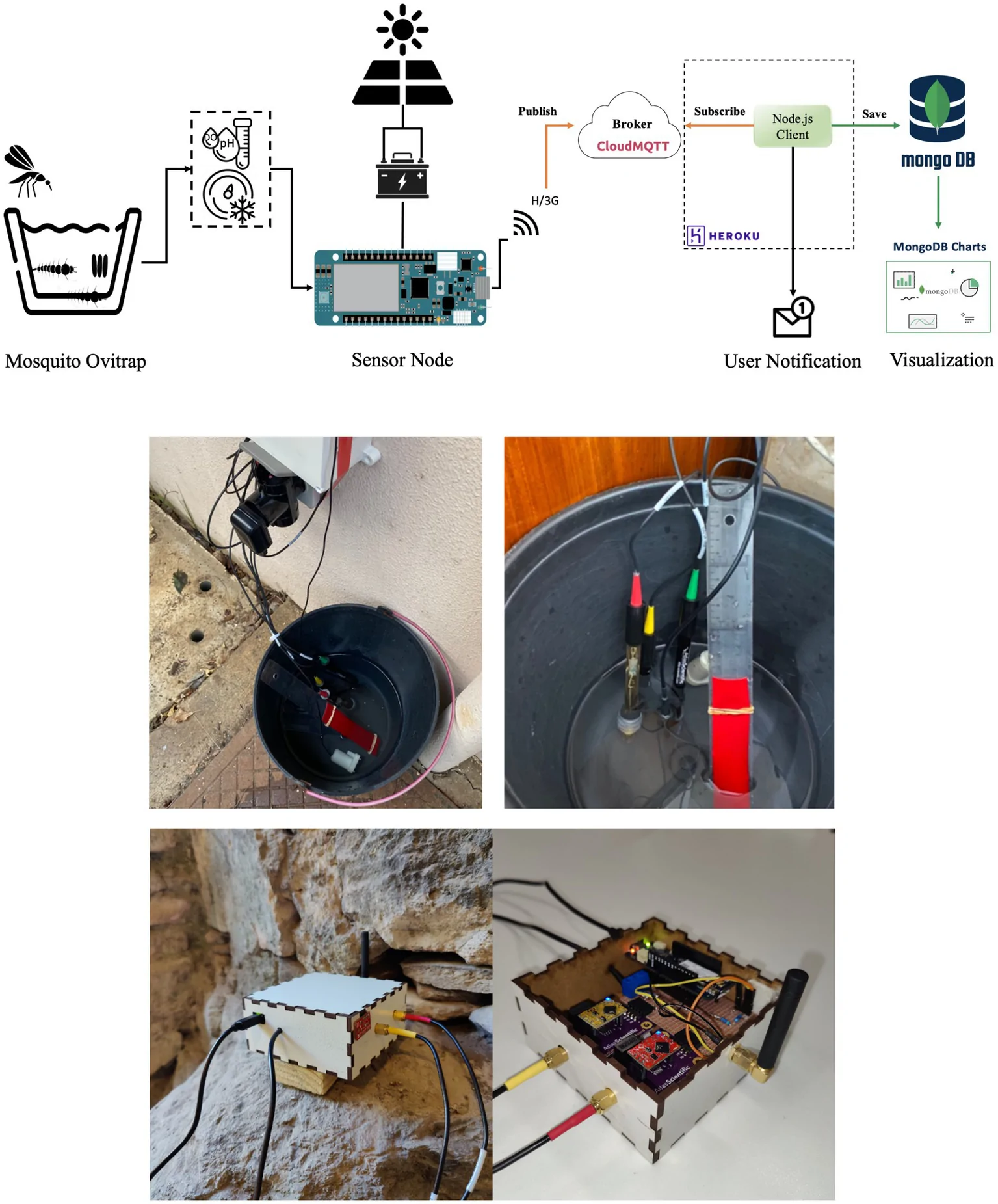
MOISS architecture. It depicts how data are continuously captured from ovitraps in the field and automatically uploaded to the cloud server (from Li et al. (30)).

Practical challenges were encountered during the deployment of the IoT technology in real-world settings in Madeira and Brazil. These included sensor failures due to technical issues, such as algae growth in Madeira and frequent power outages in Brazilian households, requiring the sensor sets to be restarted or replaced, or the affected data streams to be excluded from the analysis. Furthermore, communication outages required a switch from 3G/5G SIM networks to local Wi-Fi, where available, because of signal fluctuations. Sensor failures also occurred because of external interference, such as the accidental overturning of an ovitrap, which resulted in water loss and sensor damage. These challenges present unique insights into real-world deployment in high-income and LMIC settings, and their mitigation is described in detail in (57).

Having briefly described the VBD deployment of the OHSEW framework in Madeira and Brazil, the next four sections discuss the implementation of OHSEW Layers 1–4; Layers 5 and 6 were not required for these deployments.

### 3.2 One Health Data Sources

The project explored a number of data sources required for mosquito surveillance and early warning of breeding hotspots (45). These included environmental data, weather data, open-source data, IoT streams, longitudinal historical data, and, most importantly, the real-time mosquito surveillance data collected through the mobile apps and IoT sensors described above.

Environmental data included the following:

- Vegetation cover from US Geological Survey (USGS) EarthExplorer;
- Land-surface elevation data from the Shuttle Radar Topography Mission (SRTM) Digital Elevation Database;
- Aridity data from the GAI-PECD database; and
- Anthropogenic data from WorldPop.org, including built-up settlement, population density, nighttime lighting, and socioeconomic deprivation.

Weather data were obtained from WorldClim and the OpenWeatherMap API.

In addition to open-source data, the most important datasets were generated from the bespoke mobile surveillance apps used by environmental agents on a daily basis in the two locations, replacing paper-based systems. The app deployment in Madeira produced over 36 months of production data from 2022, whereas the deployment in Cabedelo, Brazil, produced 15 months of production data from 2024, including data from 11,576 property inspections (29, 58) and reported satisfactory usability among the agent end users.

Finally, the bespoke IoT sensor data included meteorological parameters (air temperature, humidity, and pressure) and physicochemical parameters of water (pH, dissolved oxygen, temperature, conductivity, salinity, and total dissolved solids), which were transmitted in real time to the cloud-based server via a GSM network.

In addition, through our partnership with the Regional Ministry of Health in Madeira, we received a longitudinal historical dataset comprising manually collected data on ovitrap positivity and mosquito egg abundance, as well as data from local weather stations, covering a period of over 10 years. Furthermore, we received manually collected historical data from property visits in Recife, Brazil, documenting egg, larval, and pupal infestations during the period 2009–2017.

Table 2 provides an overview of the data sources used across all pilots and deployment sites.

**Table 2:** Overview of data sources, deployment sites, and years of data collection in all pilots.

Pilots/Data streams	Madeira, Portugal	Cabedelo, Brazil	Recife, Brazil
Agents App - ovitrap entry module	Realtime data 2024–26	Realtime data from 2026	
Agents App - property entry module		Realtime data 2024–25	
Laboratory App - lab entry module	Realtime data 2024–26		
Managers Dashboard - visualise		Since 2026	
Ovitraps egg count and weather data	Historical data 2013–2026		
Environmental data (see section 4.2)	Open source 2013–2026		
IoT water and weather sensors	Realtime data 2023–2024		Installed 2024
Property visits breeding containers data			Historical data 2009–17

### 3.3 Data Integration through a Federated Architecture

For the pilot deployments, the real-time datasets were disparate in terms of temporal and spatial coverage (22, 45) and were produced daily by environmental agents at two deployment sites, Cabedelo, Brazil, and Madeira, Portugal. The data underwent cleaning by the agents and managers responsible for data entry, as well as the removal of test data by our team. Because the datasets were maintained in separate databases for data protection and security reasons, a federated architecture was not required for these deployments. As the project evolves, implementations of a federated architecture and multi-tenant database design are underway to support scalability as more municipalities commit to adopting the system in Brazil and Portugal in 2026–27.

### 3.4 Spatiotemporal and AI Modelling for Early Warning

Statistical and AI modelling were adopted in several complementary studies—the details of these methods are beyond the scope of this study and can be found in the referenced publications.

First, we developed an AI model for forecasting dengue, chikungunya, and Zika cases (59), and a climate-driven Bayesian spatiotemporal model using INLA based on a decade of historical weekly mosquito surveillance data from Madeira. The model used weather inputs to predict where and when mosquito populations would peak 2–6 weeks ahead. Elevation, urbanisation, and high tree cover were associated with spatial patterns, while seasonal temperature and precipitation were strong spatiotemporal drivers; precipitation alone was negatively associated with mosquito abundance with a 4-week lag. This study is in the final stages of submission (60).

Furthermore, using the IoT dataset from Madeira, Spearman’s rank correlation coefficient was used to analyse IoT sensor data calibrated by ground-truth observations provided by environmental agents. This analysis demonstrated statistically significant positive correlations between air temperature, dissolved oxygen, and water temperature and the presence of mosquito eggs, with correlation coefficients of 0.207 (*p*-value = 0.001), 0.289 (*p*-value < 0.001), and 0.217 (*p*-value = 0.001), respectively (57). A Bayesian hierarchical model was then used to predict egg abundance, with dissolved oxygen again demonstrating the strongest association (relative risk, 3.28; 95% CI 1.67–6.11). Finally, IoT data streams were analysed using a recurrent neural network (RNN) with LSTM, achieving a precision of 59.17%, a recall of 65.42%, and an F1 score of 58.97%. The predictive power was diluted by the inclusion of multiple development stages, including eggs, larvae, and pupae. Full details are provided in (57).

Finally, a neural network classifier was developed to predict high-risk containers as potential breeding sites in Recife, Brazil, using manually collected historical data from 2009 to 2017. An Online Extreme Learning Machine (OELM) and an OELM based on a Radial Basis Function Neural Network (OELM-RBFNN) were deployed, identifying “water tanks” and “tyres” (of seven container types surveyed) as most common water-holding containers, with average testing accuracies of 82.87% for OELM and 81.17% for OELM-RBFNN, respectively (61).

Real-time data provided by the apps in Madeira and Brazil have not yet been used for AI modelling because of the insufficient longitudinal duration of data collection and limited geographical coverage, as not all agents operating at the two sites participated in the pilot deployments. The case studies demonstrate that different aggregation windows may be appropriate for different data sources. In the IoT sensor network, an anchored window was selected, with signals sent to the cloud every 5 min unless an event triggered a more frequent response, whereas in the INLA study, a monthly sliding window was more appropriate given the granularity of the historical longitudinal data.

Integration of these models will be pursued as we continue to collect homogeneous real-time data from additional sites and extend the longitudinal coverage.

### 3.5 Component-Based Configurable Dashboard

The configurable dashboard for managers was developed, building on the previous Medi+Board surveillance and EI projects (24, 62).

The purpose of the manager and policymakers’ dashboard is to visualise the data and allow easy filtering and searching, such as searching by agent or neighborhood, visualising high-risk areas, and categorizing laboratory egg count data using heatmaps. This allows decision-makers to prioritise the highest-risk hotspots and direct agents’ efforts accordingly (Figure 6).

**Figure 6:**
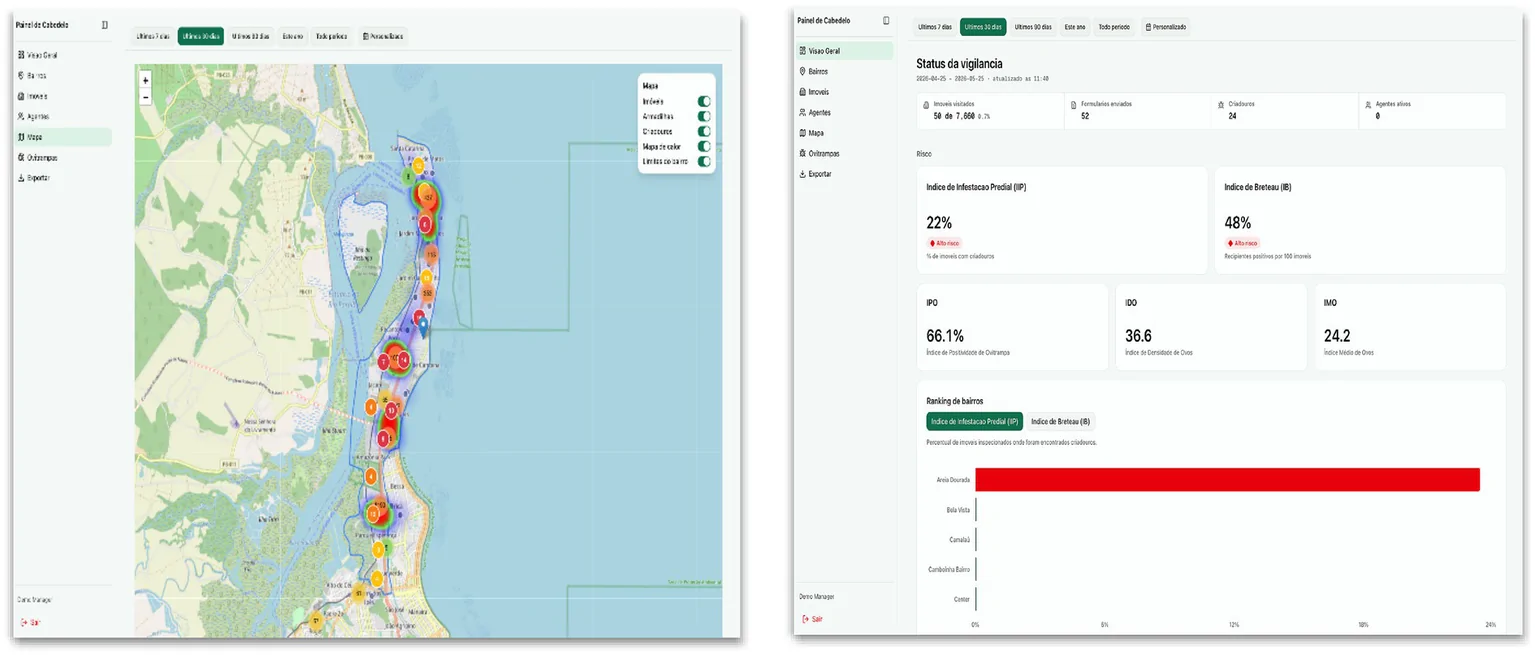
Example of dashboard for decision-makers.

As these deployments are at the pilot stage, OHSEW Layers 5 and 6 were not required and will be included in future versions of the system.

### 3.6 Network of Networks for VBD Digital Surveillance scale-up

These deployment results led to the establishment of the VBD Digital Surveillance Network of Networks (NoNs), which was launched in March 2025 in Brazil in collaboration with WHO and the Brazilian Ministry of Health. The initiative targets expansion across multiple Brazilian municipalities and integration with a VBD Network in Europe through expansion from Madeira to mainland Portugal. The NoN will support the scale-up deployment of the OHSEW system to thousands of agents in 2026–2027, further strengthening the predictive modelling capacity and generating evidence for rapid response at all decision-making levels.

### 3.7 Strengths and Limitations

Unlike typical academic projects, the app and dashboard systems have been implemented in real-world settings at two sites and have demonstrated technological maturity through continuous use over 2 years, with positive user experience. Their modular design supports easy customisation to local needs, and stakeholder feedback has also been positive. The OHSEW project was also recognised for its global impact and achievements as the runner-up in the Emerging Technology category of the Women in Tech Excellence Awards 2024 and was shortlisted for the 2026 Prix Galien Awards by the Galien Foundation.

However, there are some limitations of the current pilots, particularly in terms of data quality. First, the historical data from Recife, Brazil, for the water-container study using OELM, were only available at the district level rather than the property level, limiting the use of the results for policy-making. This limitation has now been addressed through the deployment of the app for data entry with GPS coordinates. Second, the IoT study provided promising results, but the accuracy of the neural network analysis (NNA) was modest, partly because all stages of mosquito development (eggs, larvae, and pupae) were included and because of limited and missing data resulting from deployment challenges affecting some sensors (57). A more robust, large-scale IoT sensor set is the subject of our future research. Finally, integrating all data streams from multiple deployment sites is planned with the NoN members in 2026–2027. This integration is expected to significantly strengthen the quality of the training datasets and improve the accuracy of the predictive models.

## 4 Discussion and Future Work

In this section, we will discuss the strengths of the framework to consider when implementing OHSEW, the key challenges to address, and opportunities for scale-up.

### 4.1 Strengths

The main strengths are interdisciplinarity, privacy, transparency, data standardisation and interoperability, and generalisability.

**Interdisciplinary**

The architecture will be driven by and used by public health and One Health experts, while the details of analysis, bias, and AI model training will be led by public health experts and ecologists rather than AI engineers. Through the process of adoption and localisation of the framework for their specific needs, users might prefer to tune the architecture for impact and environmental plausibility or opt for simple yet tailored solutions rather than fully generic ones with the best precision and recall.

**Privacy**

Data privacy and responsible data sharing are recommended to be built into the system and protected through open data frameworks, as defined in Layer 2, rather than treated as an afterthought (63). This is an essential matter of public trust in technology, which was severely dented after COVID-19 with the arrival of Big Tech in healthcare and governments (64).

**Data standardisation and interoperability**

Well-defined standards need to be defined and implemented in Layers 1 and 2: One Health Data and Data Integration through a Federated Architecture.

**Generalisability**

While the pilot was implemented for mosquito-borne disease surveillance, the framework and associated tools are generalisable to routine data collection for other vectors—we are currently exploring the adaptation of the digital tools for tick surveillance.

Engagement with public health stakeholders builds on the collaboration from the mosquito surveillance networks. Implementing OHSEW for human disease surveillance, such as influenza or influenza-like illness (ILI), will require engagement of public health agencies, access to and sharing of reported data, and use of data provided by participatory surveillance apps such as InfluenzaNet^2^ (popular due to the high prevalence of the disease in the community, which was not the case for VBDs). However, the components and pipeline of the OHSEW framework will remain the same.

### 4.2 Challenges and Limitations

In this section, we outline key challenges to address when building AI predictive systems using the OHSEW framework, aligned with generic principles of responsible AI (39). While a detailed discussion of these principles is beyond the scope of this study, we outline a suitable governance framework and key studies to follow.

**Explainability**

AI-generated models used in the framework cannot be non-reproducible black boxes (65); they need to be sufficiently transparent and interpretable to be trusted to support scientific learning and decision-making (42).

**Surveillance ethics and data abuse**

Ethical challenges for AI in public health surveillance have been well understood (39). These include concerns over the access to and use of citizens’ private data, such as phone mobility data (e.g., COVID-19 proximity tracking), due to the danger of disproportionate surveillance, policy measures for enforcement of compliance, unfair false positives, and documented data misuse (39, 66). However, data ownership and data access in public health (67) are societal challenges that we are currently grappling with, rather than being exclusive to One Health (68).

**Noise and misinformation**

Noise from digital data sources, fake accounts, and trolls on social media can exacerbate misinformation, making the extraction of meaningful information increasingly challenging (69). Prudence with incorporating social media (especially X) is recommended. Methods for filtering low-quality, biased news and misinformation are also recommended, as shown by an analysis of Twitter during COVID-19 (70) and around HPV vaccine misinformation (71, 72). These studies demonstrated that misinformation often originates from a small number of accounts and sources. Another major challenge for public health agencies when relying on data collected by IT providers and AI companies, rather than the bespoke tools recommended by OHSEW, is the poorly regulated role of Big Tech in health, with these companies dominating social media platforms with unprecedented political influence, gatekeeping public narratives, and having private access to healthcare systems data such as that of the NHS. OHSEW recommends building bespoke apps and tools for data collection to avoid challenges associated with open and public data sources.

These issues lead to the need for data and AI Sovereignty for Europe and other nations to reduce the overreliance on the US tech sector—for data control, AI governance, and ownership. As Europe is emerging as an important player, principles have been proposed for responsible One Health systems (73).

**Bias**

Bias in data used for training AI models is well described. This is of particular importance in One Health systems, where inequitable data collection is an inevitable outcome of the lack of resources and access in less-resourced parts of the world (74). Equitable Data Partnerships across the Global North and South are of intrinsic importance—between public health and animal health experts, ecologists, and those affected—humans, animals, farmers, and environmentalists—as well as international stakeholders. These principles are essential for interoperability in OHSEW systems integrating veterinary, medical, and environmental systems through federated architectures.

In general, more robust ethical governance for AI models is required, relying on clarifying core ethical principles from the outset of development while empowering citizens and stakeholders to participate in collaborative governance frameworks supporting ethics-by-design methodologies for systems using AI to be equitable and safe beyond One Health (75).

### 4.3 Effective Deployment and Future Work

In order to effectively deploy the OHSEW architecture in any context, national investment in infrastructure, routine surveillance, and capacity building is essential.

Furthermore, safe data-sharing platforms are required. In Europe, the open health data paradigm is supported through the European Health Data Space (EHDS) (76). EHDS establishes common standards for health data quality, interoperability, and cross-border access, and, as a result, should enable AI model training on diverse data from European populations, improving fairness. EHDS also includes provisions for secondary data use in research while maintaining strong safeguards. EHDS provides a new opportunity for One Health systems.

In terms of AI responsibility and safety, the use of AI models developed and trained for specific diseases by researchers at the national level might be the natural choice. If industry models are considered by national governments, their governance might be a challenge, as the recent WHO report highlighted (77). Even in the European region, only 8% of MSs have an AI vision and health strategy; AI literacy is lagging behind technological developments, and EHDS readiness remains low (only 30% have guidance on the secondary use of health data and cross-border sharing) (78).

Another challenge lies in the intrinsically collaborative nature of public health and One Health and the need for the implementation of data standards and interoperability, as highlighted in OHSEW Layer 2: Data Integration through a Federated Architecture. This is much more challenging in public health and One Health settings than in primary and clinical care, which is a national responsibility and where data standards are normally well regulated (79). One Health relies on multiple international organisations to work together and agree on standards, data sharing, model training, and coordinating response.

Our current efforts and future research include further recommenders on the selection of One Health data streams for various diseases (Layer 1), standards and protocols (Layer 2), selection of models (Layer 3), and definitions of components for the dashboard (Layer 4).

## 5 Conclusion

Pandemic preparedness and global health security rely on One Health—the interaction among humans, animals, and the environment. However, leveraging the abundance of real-time, geolocated data sources and developing bespoke apps and digital tools for AI predictive modelling and IoT environmental monitoring, trained and calibrated by routine surveillance data for One Health needs, remain unfulfilled.

In this study, we presented the OHSEW framework—a human-centric architecture for One Health surveillance, early warning, and response leveraging heterogeneous data sources for AI modelling and IoT environmental monitoring, championing the development and deployment of bespoke mobile tools and IoT sensor sets to fill the gap in missing fine-grained, real-time, GPS-enabled surveillance datasets, particularly much-needed ones in LMICs. The architecture consists of six layers implementing the data pipeline—One Health Data Sources, Data Integration through a Federated Architecture, Spatiotemporal and AI Modelling for Early Warning, Component-based Configurable Dashboards for decision-makers, Interventions and Control Measures, and Public Risk Communication and Community Engagement.

We have demonstrated the feasibility of the framework through a real-word pilot for mosquito surveillance deployed with users in Brazil and Madeira over 2 years, demonstrating the potential of the framework in real-world settings.

## Data Availability

The original contributions presented in the study are included in the article/supplementary material, further inquiries can be directed to the corresponding author.

## Footnotes

1
https://www.who.int/initiatives/eios

2
https://influenzanet.info/home
